# Pharmacogenomic Diversity among Brazilians: Influence of Ancestry, Self-Reported Color, and Geographical Origin

**DOI:** 10.3389/fphar.2012.00191

**Published:** 2012-11-06

**Authors:** Guilherme Suarez-Kurtz, Sergio Danilo Juno Pena, Claudio José Struchiner, Mara Helena Hutz

**Affiliations:** ^1^Programa de Farmacologia, Coordenação de Pesquisa, Instituto Nacional de CâncerRio de Janeiro, Brazil; ^2^Departamento de Bioquímica e Imunologia, Universidade Federal de Minas GeraisBelo Horizonte, Brazil; ^3^Programa de Computação Científica, Fundação Oswaldo CruzRio de Janeiro, Brazil; ^4^Departamento de Genética, Universidade Federal do Rio Grande do SulPorto Alegre, Brazil

**Keywords:** biogeographical ancestry, Brazilian pharmacogenomic network, *F*_ST_ statistics, pharmacogenomic diversity, population admixture, refargen

## Abstract

By virtue of being the product of the genetic admixture of three ancestral roots: Europeans, Africans, and Amerindians, the present-day Brazilian population displays very high levels of genomic diversity, which have important pharmacogenetic/-genomic (PGx) implications. Recognition of this fact has prompted the creation of the Brazilian Pharmacogenomics Network (Refargen), a nationwide consortium of research groups, with the mission to provide leadership in PGx research and education in Brazil, with a population heath impact. Here, we present original data and review published results from a Refargen comprehensive study of the distribution of PGx polymorphisms in a representative cohort of the Brazilian people, comprising 1,034 healthy, unrelated adults, self-identified as white, brown, or black, according to the Color categories adopted by the Brazilian Census. Multinomial log-linear regression analysis was applied to infer the statistical association between allele, genotype, and haplotype distributions among Brazilians (response variables) and self-reported Color, geographical region, and biogeographical ancestry (explanatory variables), whereas Wright’s *F*_ST_ statistics was used to assess the extent of PGx divergence among different strata of the Brazilian population. Major PGx implications of these findings are: first, extrapolation of data from relatively well-defined ethnic groups is clearly not applicable to the majority of Brazilians; second, the frequency distribution of polymorphisms in several pharmacogenes of clinical relevance (e.g., *ABCB1*, *CYP3A5*, *CYP2C9*, *VKORC*) varies continuously among Brazilians and is not captured by race/Color self-identification; third, the intrinsic heterogeneity of the Brazilian population must be acknowledged in the design and interpretation of PGx studies in order to avoid spurious conclusions based on improper matching of study cohorts.

## Introduction

The present-day Brazilian population, in excess of 190 million people, is highly heterogeneous and admixed, as result of five centuries of mating between native Amerindians, Europeans, and sub-Saharan Africans. This fact renders inappropriate extrapolation of pharmacogenetic/-genomic (PGx) data derived from well-defined ethnic groups to the majority of Brazilians. Recognition of this fact has prompted the creation of the Brazilian Pharmacogenomics Network or Refargen (Suarez-Kurtz, 2004), a nationwide consortium of research groups, mostly from academia^1^. In consonance with its mission to provide leadership in PGx research and education in Brazil, with impact on population heath (Suarez-Kurtz, 2009), Refargen has recently concluded a comprehensive study of the distribution of PGx polymorphisms among Brazilians. In this article, we will present original data and review previously published results (Suarez-Kurtz et al., 2010, 2012a,b,c; Pena et al., 2011; Sortica et al., 2012) from the Refargen study and discuss the PGx implications of the findings for Brazilians and possibly other admixed populations of the Americas.

**Figure 1:**
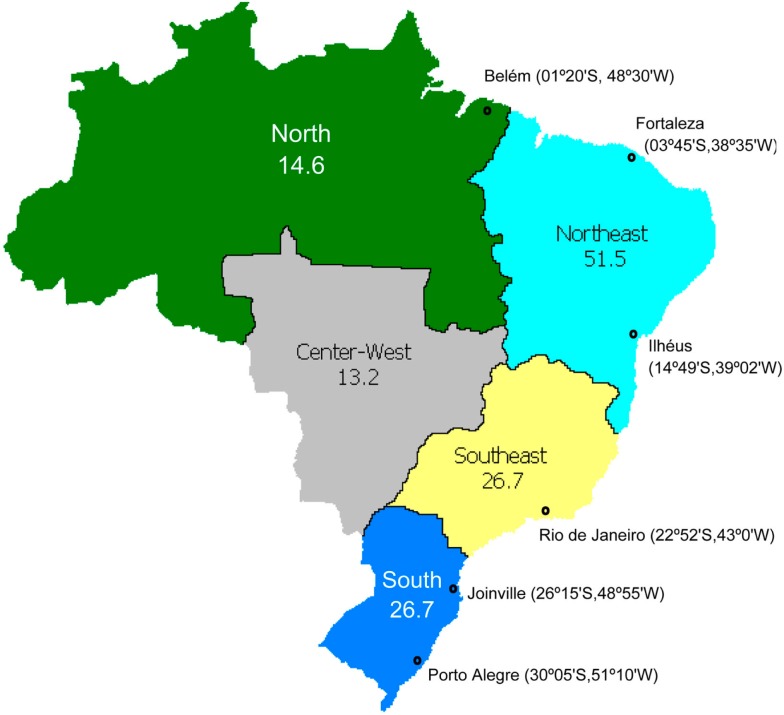
**Map of Brazil, showing its five geographical regions, their populations (in millions), and cities where individuals were recruited for the Refargen study, reviewed in this article**.

The study cohort consisted of 1,034 healthy, unrelated adults recruited in the North, Northeast, Southeast, and South regions of Brazil (Figure 1). Each individual signed a written informed consent and was asked to self-identify according to the classification scheme adopted by the Brazilian Census^2^, which relies on self-perception of skin color. Accordingly, the subjects were distributed into three groups: *branco* (White, *n* = 342), *pardo* (Brown, *n* = 350), and *preto* (Black, *n* = 342). The term Color is capitalized throughout the text, to call attention to its special meaning in the context of the Brazilian Census classification. This cohort is considered representative of the present-day Brazilian population since 99% of Brazilians self-identify in one of the three Color categories, and 93% live in one of the four regions, included in the study^3^. Individuals from the Center-West region (7% of the Brazilian population) and those classified as “Yellow” (meaning Asian descendants, 0.7%) or Amerindian (0.3%) were not included in the study. We genotyped 44 loci in 12 pharmacogenes (Table 1) which modulate drug metabolism (*CYP2B6*, *CYP2C8*, *CYP2C9*, *CYP2C19*, *CYP2D6*, *CYP3A5*, *COMT*, and *TPMT*), transport (*ABCB1*, *SLCO1B1*, and *SLCO1B3*) and effect (*VKORC1*). Pharmacogenomics Knowledge Base (PharmGKB^4^) lists all these genes, except *SCLO1B3*, as “Important PGx genes (VIP)” and two thirds of the 44 polymorphisms investigated as “Important Variants.”

**Table 1:** **Distribution of pharmacogenetic polymorphisms among Brazilians**.

Gene	Polymorphism	Id #	Effect	Minor allele frequency				Chi square	*F*_ST_		
				Overall	White	Brown	Black	*P* value	White vs. Black	White vs. Brown	Brown vs. Black
*ABCB1*	1267C > T	rs1128503	G412G	0.380	0.412	0.362	0.312	0.18	0.011	0.003	0.003
	2677G > T/A	rs2032582	S193A/T	0.370	0.417	0.343	0.221	**0.0006**	0.044	0.006	0.019
	3435C > T	rs1045642	I1145I	0.427	0.458	0.411	0.317	**0.037**	0.021	0.002	0.009
*COMT*	472G > A	rs4680	V158M	0.408	0.437	0.374	0.411	0.52	0.001	0.004	0.001
*CYP2B6*	64C > T	rs8192709	R22C	0.081	0.067	0.099	0.078	0.60	0.001	0.003	0.001
	785A > G	rs2279343	K262R	0.303	0.295	0.300	0.374	0.22	0.007	0.000	0.006
	1459C > T	rs3211371	R487C	0.172	0.213	0.135	0.127	0.068	0.013	0.011	0.000
	516G > T	rs3745274	Q172H	0.369	0.393	0.325	0.458	0.055	0.004	0.005	0.019
*CYP2C8*	805A > T	rs11572103	I269F	0.047	0.026	0.060	0.106	**0.012**	0.026	0.007	0.007
	416G > A	rs11572080	R139K	0.098	0.125	0.075	0.058	0.08	0.014	0.007	0.001
	792C > G	rs1058930	I264M	0.038	0.040	0.039	0.020	0.54	0.004	0.000	0.003
*CYP2C9*	430C > T	rs1799853	R144C	0.108	0.137	0.082	0.066	0.08	0.004	0.001	0.007
	1075A > C	rs1057910	I359L	0.052	0.049	0.059	0.026	0.26	0.014	0.008	0.001
	1080C > G	rs28371686	D360E	0.005	0.004	0.005	0.009	1.0	0.001	0.000	0.000
	1003C > T	rs28371685	R335W	0.008	0.010	0.005	0.009	0.78	0.000	0.001	0.000
*CYP2C19*	681G > A	rs4244285	Splicing defect	0.115	0.106	0.120	0.144	0.58	0.003	0.001	0.001
	636G > A	rs4986893	W212X	0.000	0.000	0.000	0.003	1.0	0.000	0.000	0.000
	−806C > T	rs12248560	Increased transcription	0.169	0.168	0.167	0.175	0.98	0.000	0.000	0.000
*CYP2D6*	−1584C > G	rs1080985	Promoter region	0.218	0.235	0.207	0.178	0.64	0.003	0.003	0.000
	31G > A	rs769258	V11M	0.035	0.043	0.027	0.023	0.60	0.002	0.002	0.000
	100C > T	rs1065852	P34S	0.136	0.150	0.124	0.115	0.79	0.003	0.002	0.000
	1023C > T	rs28371706	T107I	0.044	0.021	0.057	0.111	0.08	0.025	0.011	0.003
	1661G > C	rs1058164	V136V	0.456	0.461	0.445	0.482	0.89	0.000	0.000	0.001
	1846G > A	rs3892097	Splicing defect	0.117	0.134	0.105	0.080	0.61	0.003	0.004	0.000
	2549A > del	rs35742686	Frame shift	0.009	0.011	0.007	0.006	0.60	0.000	0.000	0.000
	2615-2617delAAG	rs5030656	K281del	0.013	0.014	0.011	0.013	1.0	0.001	0.002	0.001
	2850C > T	rs16947	R296C	0.390	0.352	0.417	0.480	0.23	0.016	0.003	0.005
	3183G > A	rs59421388	V287M	0.026	0.011	0.038	0.056	0.25	0.021	0.011	0.002
	4180G > C	rs1135840	S486T	0.531	0.513	0.541	0.582	0.74	0.004	0.000	0.002
*CYP3A5*	6986A > G	rs776746	Frame shift	0.698	0.785	0.627	0.541	**<0.0001**	**0.067**	0.030	0.007
	14690G > A	rs10264272	Splicing defect	0.027	0.004	0.039	0.105	**0.0001**	0.049	0.014	0.017
	23132insT	rs413003343	Frame shift	0.024	0.013	0.027	0.079	**0.003**	0.025	0.003	0.013
*SLCO1B1*	388A > G	rs2306283	N130D	0.553	0.498	0.601	0.635	**0.036**	0.019	0.011	0.001
	463C > A	rs11045819	P155T	0.118	0.118	0.122	0.097	0.77	0.001	0.000	0.002
	521T > C	rs4149056	V174A	0.135	0.135	0.143	0.089	0.24	0.005	0.000	0.007
*SLCO1B3*	334T > G	rs4149117	S112A	0.741	0.799	0.702	0.592	**0.0003**	**0.051**	0.013	0.013
	699G > A	rs7311358	M233I	0.741	0.799	0.702	0.592	**0.0003**	**0.051**	0.013	0.013
*TMPT*	238G > C	rs1800462	A80P	0.008	0.001	0.014	0.014	0.36	0.006	0.006	0.000
	460G > A	rs1800460	A154T	0.010	0.009	0.011	0.010	0.82	0.000	0.000	0.000
	719A > G	rs1142345	Y240C	0.026	0.017	0.037	0.017	0.46	0.000	0.004	0.004
*VKORC1*	3673G > A	rs9923231	Reduced transcription	0.333	0.371	0.306	0.238	**0.038**	0.021	0.005	0.006
	5808C > T	rs2884737	Intronic	0.193	0.255	0.135	0.128	**0.003**	0.026	0.023	0.000
	6853G > C	rs8050894	Intronic	0.392	0.404	0.386	0.357	0.65	0.002	0.000	0.001
	9104G > A	rs7294	3-UTP	0.375	0.376	0.372	0.379	0.65	0.000	0.000	0.000

*Bold numbers in the column “chi square” indicate statistically significant *P* values; bold numbers in the “White vs. Black” column indicate moderate pharmacogenetic divergence*.

**Figure 2:**
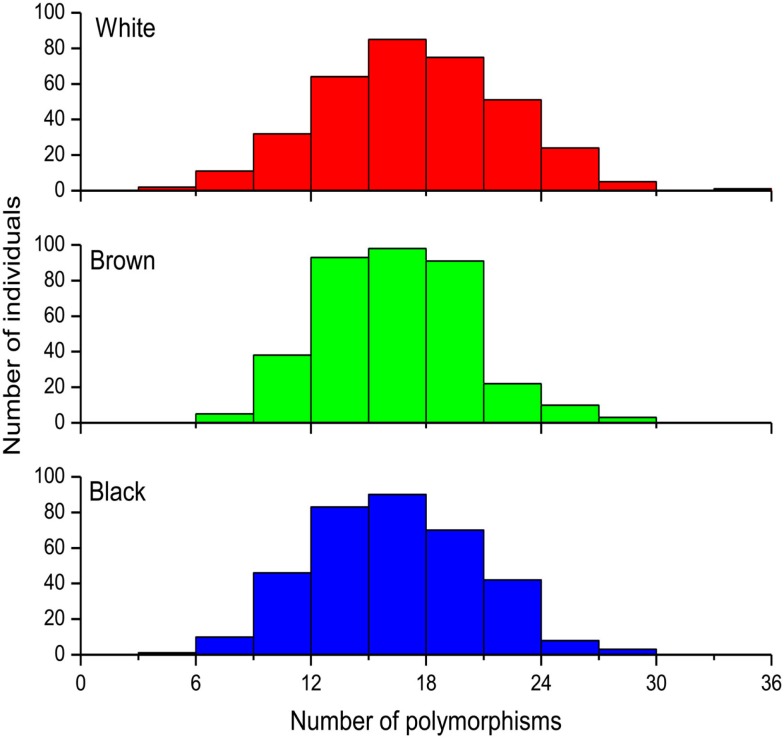
**Frequency histograms of the distribution of 44 PGx polymorphisms among self-identified (White *n* = 342), Brown (*n* = 352), and Black Brazilians (*n* = 342)**. The data represent the total number of minor alleles at the 44 loci, in each individual.

We will initially present data for the overall cohort and for each Color group within this cohort. Figure 2 shows frequency histograms of the total number of minor alleles identified in each individual. No statistically significant difference (Kruskal–Wallis test *p* = 0.92) was detected across the three Color groups, the median (interquartile range) number of polymorphisms being 17 (14–20), 16 (13–18), and 16 (13–19) in White, Brown, and Black individuals. This adds to 18.9% of the total number of alleles genotyped at the 44 loci in the overall cohort. However, the allele frequency at 11 of these loci differed significantly (chi square *p* < 0.05) across the Color groups. The pharmacogenes affected were *ABCB1* (2 SNPs), *CYP2C8* (1) *CYP3A5* (3), *NAT2* (3), *SLCO1B1* (1), *SCLO1B3* (2 SNPs, which are in complete LD) and *VKORC1* (2). We applied the Wright’s *F*_ST_ statistics (Wright, 1951) to estimate the extent of PGx divergence among the three Color strata, and observed mean *F*_ST_ values of 0.005 (SD 0.006), 0.013 (0.017), and 0.004 (0.005) for pair-wise comparisons of White vs. Brown, White vs. Black, and Brown vs. Black, respectively (Suarez-Kurtz et al., 2012b). According to Wright’s qualitative guidelines (Wright, 1978), *F*_ST_ values lower than 0.05 denote low genetic diversity, whereas values between 0.05 and 0.15 indicate moderate diversity. As shown in Table 1, only three SNPs, namely CYP3A5*3 and the linked *SLCO1B3* 334T > C and 699G > A transitions exceeded, and two other SNPs (*ABCB1* 2677G > nonG and CYP3A5*6) approached, the *F*_ST_ threshold for moderate genetic divergence in White vs. Black Brazilians in the entire cohort. Not surprisingly, these were the SNPs with the smallest *p* values for the Kruskal–Wallis analyses of frequency distribution in the overall cohort (<0.0001–0.0006, Table 1). Taken together, the *F*_ST_ analyses in the overall cohort suggest low PGx divergence at all loci interrogated in self-identified Brown vs. White or Black individuals, whereas moderate divergence was observed at three, and possibly five loci (out of the 44 investigated) in pair-wise comparisons of White vs. Black Brazilians.

## Distribution of Pharmacogenetic Polymorphisms among Brazilians According to Color Categories and Geographical Regions

With an area of 8,511,960 Km^2^, Brazil is a country of continental size (the fifth largest in the world) and different regions have diverse population histories. For instance, the North had a large influence of the Amerindian root, the Northeast had a history of strong African presence due to slavery and the South was mostly settled by European immigrants (Pena et al., 2011). We have applied multinomial log-linear regression analyses (Suarez-Kurtz et al., 2010, 2012c; Sortica et al., 2012) to infer the statistical association between allele, genotype, and haplotype distributions among Brazilians (response variables) and self-reported Color and geographical region (explanatory variables). This procedure obviates the need for correction for multiple comparisons, because the main effects and interaction terms are tested simultaneously within each regression context. Table 2 illustrates results from this exercise, applied to selected genes affecting drug metabolism (*CYP2C8*, *CYP2C9*, and *CYP2C19*), transport (*ABCB1* and *SLCO1B1*) and response (*VKORC1*). Color *per se* associates significantly with the frequency distribution of *CYP2C8* and *CYP2C9* variant alleles, *ABCB1* and *SLCO1B1* haplotypes, and *VKOC1 3673G* > *A* alleles and genotypes; no association is observed with respect to the *CYP2C19* polymorphisms. Color in combination with geographical region is significantly associated with distribution of *CYP2C8* and *CYP2C9* alleles, *ABCB1* and *SLCO1B1* haplotypes, whereas geographical region *per se* associates with *CYP2C8* and *CYP2C9* allele frequency.

**Table 2:** **Multinominal log-linear analyses of the distribution of pharmacogenetic polymorphisms alleles among Brazilians according to self-reported color and geographical region**.

Gene	Alleles	Explanatory variables^a^		
		Color	Geographical region	Color: geographical region
*CYP2C8*	*2, *3, *4	<0.0001	0.71	0.04
*CYP2C9*	*2, *3, *5, *11	<0.0001	0.23	0.01
*CYP2C19*	*2, *3, *17	0.60	0.11	0.33
*ABCB1*	haplotypes^b^	<0.001	0.001	0.013
*SLCO1B1*	haplotypes^c^	<0.001	0.001	0.003
*VKORC1*	3673A allele	0.0004	0.07	0.11
	3673A genotype	0.002	0.18	0.19

^a^*p* values associated to the “main effects” (Color and geographical region) and their “interaction.”

*^b^Haplotypes comprising the 1236C > T, 2677G > nonG, and 3435C > T loci*.

*^c^Haplotypes comprising the 388A > G, 463C > A, and 521T > C loci*.

**Figure 3:**
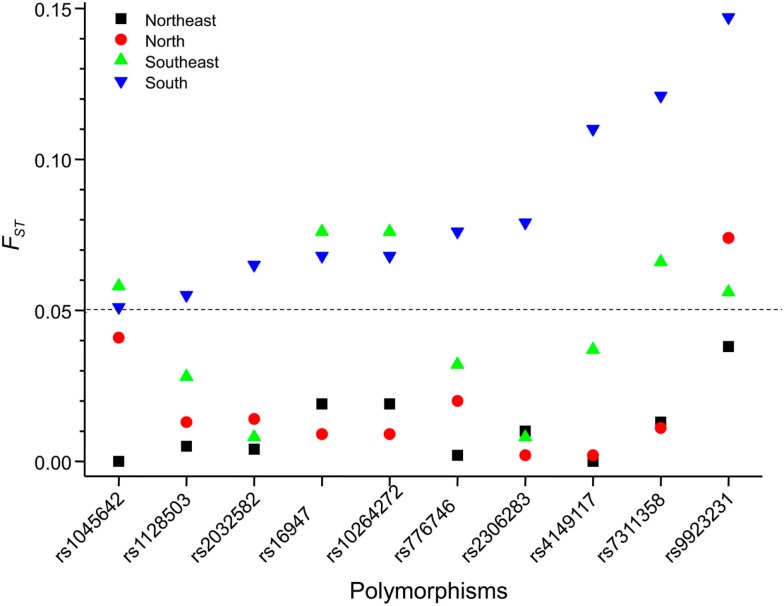
**Allele-specific *F*_ST_ values for 10 PGx polymorphisms (*x*-axis) in White vs. Black Brazilians recruited at the North, Northeast, Southeast, and South regions**. The dashed line shows the threshold *F*_ST_ values (0.05) for moderate genetic divergence. The genes and loci for each polymorphism are presented in Table 1.

**Figure 4:**
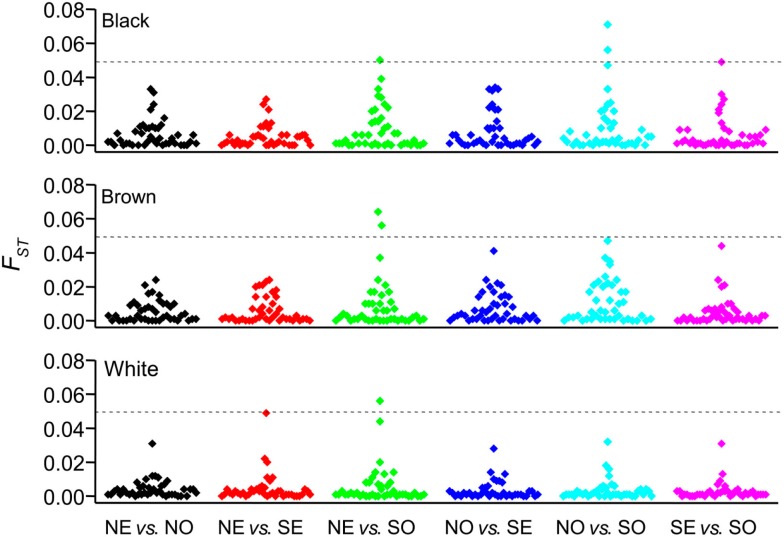
**Allele-specific *F*_ST_ values (*y*-axis) for pair-wise comparisons between geographical regions (*x*-axis)**. Data are presented separately for self-identified Black (top panel), Brown (middle panel), and White (bottom panel) individuals. Each symbol correspond to one of the 44 polymorphisms listed in Table 1. The dashed line shows the threshold *F*_ST_ values (0.05) for moderate genetic divergence. NO, North; NE, Northeast; SE, Southeast; SO, South.

We explored further the PGx heterogeneity among Brazilians by the *F*_ST_ statistics. First, we performed pair-wise comparisons between Color groups within each geographical region, and detected significant differences in the distribution of *F*_ST_ values for White vs. Brown (*P* < 0.0001, ANOVA) and White vs. Black (*P* < 0.0001), but not Brown vs. Black individuals, across regions (Suarez-Kurtz et al., 2012b). This implies that the extent of pharmacogenetic divergence between Whites and Non-Whites (i.e., Black and Brown individuals) varies significantly among regions. The data presented in Figure 3 supports this interpretation: we show that 10 selected polymorphisms in *ABCB1*, *CYP2D6*, *CYP3A5*, *SLCO1B1*, *SCLO1B3*, and *VKORC1* display moderate divergence between Whites and Blacks in the South, compared to five, one, and zero in the Southeast, North, and Northeast, respectively. In a second exercise, we compared *F*_ST_ values for each Color between regions and present the results in Figure 4. Of the 792 (44 polymorphisms × six pair-wise regions × three Color groups) comparisons, only three SNPs among Black, one among Brown, and one among White individuals exceeded the threshold (*F*_ST_ = 0.05) for moderate PGx divergence. Taken together, these *F*_ST_ results extend the conclusions of the multinomial analyses described above, that the distribution of PGx polymorphisms among Brazilians is influenced by self-reported Color, geographical region, and the interaction of these two variables. Collectively, these data reflect the notorious heterogeneity of the Brazilian population and highlight the inappropriateness of ascribing PGx polymorphisms’ frequencies for “Brazilians” based on data from one or more Color strata recruited at a given region (or city).

## Impact of Biogeographical Ancestry on the Distribution of Pharmacogenetic Polymorphisms among Brazilians

**Figure 5:**
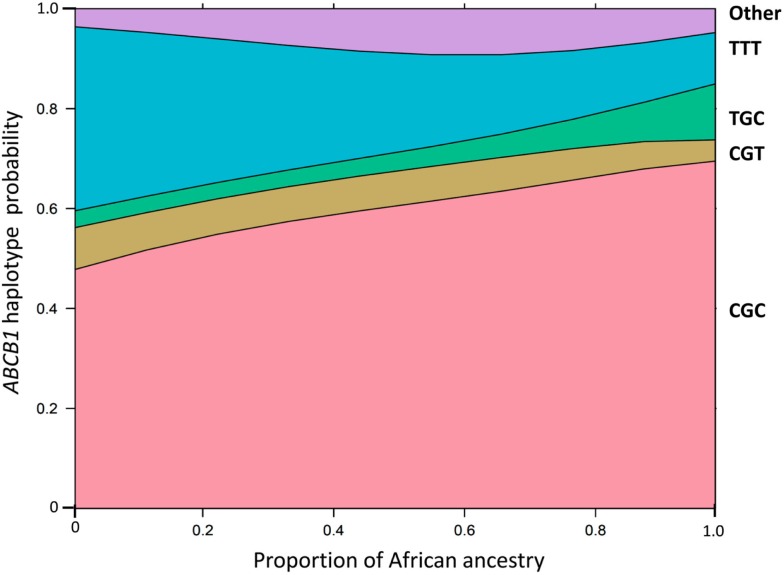
**Effect display for the distribution of *ABCB1* haplotypes in the logit model fit to the data for African ancestry in 965 Brazilians**. The haplotypes comprising the 1236C > T, 2677G > nonG, and 3435C > T SNPs are shown at the right of the plot. The individual proportion of African ancestry is shown in the *x*-axis. The *y*-axis is labeled on the probability scale. The plot was generated as described by Venables and Ripley (2002) and implemented as function “multinom” available in the R package “nnet.” Data from Sortica et al. (2012).

**Figure 6:**
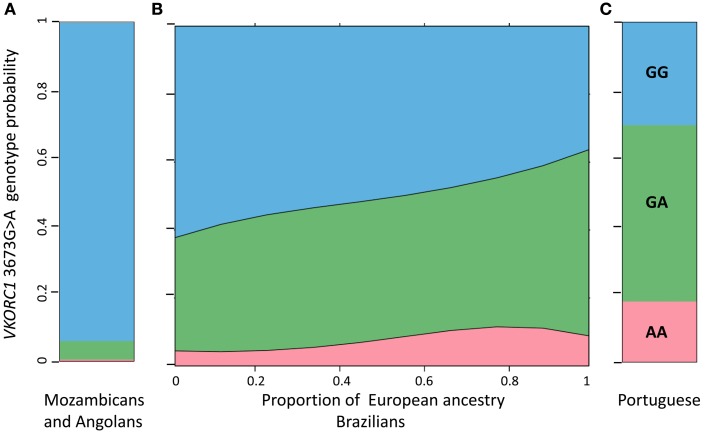
**Effect display for the distribution of *VKORC1* 3673G > A genotypes in the logit model fit to the data for African ancestry in 965 Brazilians (B)**. For comparison the frequency of each genotype in a cohort of Angolans and Mozambicans [*n* = 216, **(A)**] and in a Portuguese cohort [*n* = 89, **(C)**] are also shown. The individual proportion of African ancestry in Brazilians is shown in the *x*-axis. The *y*-axis represents the genotype probability for Brazilians and the observed genotype frequency for the African and Portuguese cohorts. Data from Suarez-Kurtz et al. (2010). The plot for Brazilians was generated as described in Figure 5.

These analyses were based on the individual proportions of European, African, and Amerindian ancestry, estimated using a panel of short insertion/deletion polymorphisms, validated as ancestry-informative markers (Bastos-Rodrigues et al., 2006), and the STRUCTURE clustering software (Pritchard et al., 2000). These data, available for 965 subjects confirmed that the vast majority of Brazilians, irrespective of self-reported Color, share European and African ancestries in variable proportions, and a sizable number of individuals display also distinct Amerindian ancestry (Suarez-Kurtz and Pena, 2006, 2007; Suarez-Kurtz et al., 2010; Pena et al., 2011). The average proportions of European ancestry decrease progressively from self-reported White (mean 0.80, SD 0.21, *n* = 325), to Brown (0.62, 0.29, 322) and then to Black individuals (0.46, 0.20, 318), and the opposite trend is observed with respect to African ancestry, which averaged 0.10 (SD 0.14) in White, 0.25 (0.26) in Brown, and 0.42 (0.29) in Black persons. However, the individual proportions of European and African ancestry varies widely, and most importantly, as a continuum within each of these three Color categories, whereas the individual proportion of Amerindian ancestry remains relatively constant across the three groups, ranging from 0.10 to 0.13. To describe the association between PGx polymorphisms and the estimated individual biogeographical ancestry we fitted non-linear logistic regression modeling using maximum likelihood estimation. A consistent finding in these analyses (Suarez-Kurtz et al., 2007a,b, 2010, 2012c; Estrela et al., 2008; Vargens et al., 2008) is that the frequency distribution of PGx polymorphisms among Brazilians is best fit by continuous functions of the individual proportions of African and European ancestry. This is illustrated in Figures 5 and 6. In Figure 5 we show that the probability of having the wild-type (C/G/C) and the T/G/C *ABCB1* haplotypes increases continuously with the increase in African ancestry, whereas the opposite trend is observed for the T/nonG/T haplotype. Figure 6 shows that the odds of having the heterozygous, and to a lesser extent, the homozygous variant genotype at the *VKORC1* 3673G > A locus increase progressively as the individual proportion of European ancestry increases. For comparison, we also display in Figure 6 the distribution of *VKORC1* 3673G > A genotypes among Portuguese, by far the most important source of European migrants from Brazil, and individuals from Angola and Mozambique, two former Portuguese colonies in Africa, and origin of enslaved Africans brought to Brazil.

**Figure 7:**
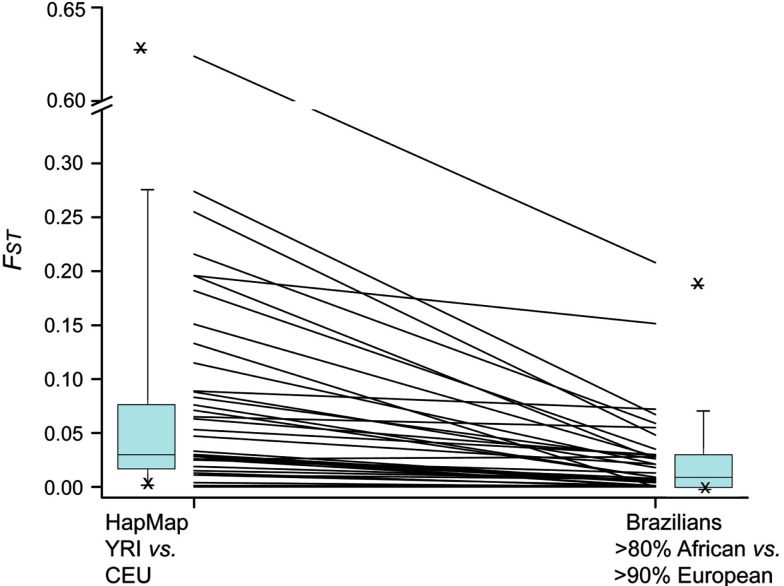
**Allele-specific *F*_ST_ values for 38 PGx polymorphisms in HapMap CEU vs. YRI and in Brazilians with >90% European ancestry vs. Brazilians with >80% African ancestry**. The lines connect the *F*_ST_ values for each polymorphism in the two data sets and the box plots at the left and right summarize the ensemble of the data for each set.

Considering that the European and African components together account for 89% of the diversity in individual genetic ancestry in the Refargen cohort (Pena et al., 2011), it might be anticipated that: (a) the greater the difference in frequency of a given polymorphism between Europeans and sub-Saharan Africans, the wider the range of frequency variation among Brazilians; (b) the range of variation among Brazilians will be smaller than the difference in frequency between Europeans and Africans, because of the admixture of these ancestral roots in Brazilians. We have previously verified both these predictions for polymorphisms in *VKORC1* (Suarez-Kurtz et al., 2010) and within the *CYP2C* cluster (Suarez-Kurtz et al., 2012c). We applied the *F*_ST_ statistics to examine these predictions in 38 polymorphisms which were genotyped in the Refargen cohort and also in the HapMap project. In Figure 7 we shown the pair-wise *F*_ST_ values for each polymorphism in HapMap CEU vs. YRI cohorts – taken as proxies of the European and sub-Saharan African ancestral roots of Brazilians, respectively – and Brazilians with >90% European ancestry vs. Brazilians with >80% African ancestry. The attenuation of pharmacogenetic divergence between the Brazilian groups compared to the HapMap populations is evident.

## Concluding Remarks and Perspectives

The kaleidoscopic diversity of the admixed Brazilian population, with tri-hybrid biogeographical ancestry in Europe, Africa, and America adds complexity to, but also creates advantages for PGx research. Advantages include the opportunity to explore PGx associations in individuals with heterogeneous genetic ancestry under similar environmental and socio-economical conditions, and to gather information on peoples that are excluded or under-represented in clinical drug trials, such as sub-Saharan Africans and Native Americans. A major challenge to PGx studies in Brazil is population stratification, which if not controlled for, will confound the outcomes of PGx association studies. Our studies describe ways to control for this caveat, by combining ancestry-informative markers and appropriate statistical approaches. A distinct message that emerges from these studies is that race/color categorization does not capture the distribution of PGx polymorphisms among Brazilians, which is best modeled by continuous functions of the individual proportions of European and African ancestry, irrespective of self-identified Color (Suarez-Kurtz, 2010). Recognition of this fact is important in the design and interpretation of PGx clinical trials in Brazilians but does not imply that PGx-informed drug prescription requires investigation of individual ancestry. Rather, individual genotyping should be directed to PGx polymorphisms of proven clinical utility for the specific medical condition being treated, irrespective of biogeographical ancestry.

Drug assessment and regulatory processes in Brazil are carried out by the National Health Surveillance Agency, ANVISA, an independently administered, financially autonomous agency, managed by a Collegiate Board of Directors^5^. ANVISA has the mandate to grant, and withdraw, product registration permits within its areas of activity, which comprise medicines for human use. Registration of new medicines do not require, that clinical trials be carried out in the Brazilian population, and evaluation of the medicine’s efficacy and toxicity is based mainly, if not exclusively, on foreign data. Despite the increasing enrolment of non–Caucasian subjects in global drug development programs, most data submitted to ANVISA derive from white Europeans and North Americans. We have recently shown that there is little pharmacogenetic divergence between the HapMap CEU cohort of European extraction and White Brazilians, such that only CYP3A5*3 among 44 polymorphisms exceeded the *F*_ST_ threshold for moderate divergence. By contrast, *F*_ST_ analyses revealed very large divergence between CEU and Black Brazilians for CYP3A5*3 and moderate divergence for eight other polymorphisms, including another *CYP3A5* SNP (CYP3A5*6) and SNPs in the *ABCB1*, *SLCO1B3*, and *SLCO1B1* genes. These findings represent a caveat against extrapolation of PGx data from European-derived (“Caucasian”) cohorts to the ensemble of Brazilians.

Admixture is common in all developing nations in the American continent, although the relative contributions of the three major ancestral roots – native American, European, and sub-Saharan African – vary among these nations, as well as among ethnic groups and geographical regions within a given country. Hence, extrapolation of conclusions drawn from PGx studies in Brazilians to other admixed Latin American populations must take into account the specific patterns of population structure and diversity across the Americas. Therapeutic drugs are usually developed and investigated for their safety and efficacy in geographical and ethnical populations that do not encompass the diversity of Latin American peoples. Drivers and barriers to the adoption of PGx in developing countries, and specific ways in which these countries could benefit from PGx-based drug therapy deserve greater attention from academic and industrial scientists, prescribers, and legislators in developing nations across the Americas. This goal is not likely to be achieved simply by mandates to include subjects from ethnic minorities in clinical drug trials, especially when these groups are labeled by phenotypes which do not accurately reflect genetic ancestry (Suarez-Kurtz, 2005, 2010; Suarez-Kurtz and Pena, 2006, 2007).

## Conflict of Interest Statement

The authors declare that the research was conducted in the absence of any commercial or financial relationships that could be construed as a potential conflict of interest.

## Footnotes

^1^http://www.refargen.org.br

^2^http://www.ibge.gov.br

^3^http://www.sidra.ibge.gov.br/bda/tabela/listabl.asp?z=t&c=26

^4^http://www.pharmgkb.org/search/browseVip.action?browseKey=annotatedGenes

^5^http://www.anvisa.gov.br/eng/index.htm
